# Investigation of an unrecognized large-scale outbreak of *Candida parapsilosis* sensu stricto fungaemia in a tertiary-care hospital in China

**DOI:** 10.1038/srep27099

**Published:** 2016-06-02

**Authors:** He Wang, Li Zhang, Timothy Kudinha, Fanrong Kong, Xiao-Jun Ma, Yun-Zhuo Chu, Mei Kang, Zi-Yong Sun, Ruo-Yu Li, Kang Liao, Juan Lu, Gui-Ling Zou, Meng Xiao, Xin Fan, Ying-Chun Xu

**Affiliations:** 1 Department of Clinical Laboratory, Peking Union Medical College Hospital, Chinese Academy of Medical Sciences, Beijing, China; 2 Graduate School, Peking Union Medical College, Chinese Academy of Medical Sciences, Beijing, China; 3 Charles Sturt University, Leeds Parade, Orange, New South Wales, Australia; 4 Centre for Infectious Diseases and Microbiology Laboratory Services, Westmead Hospital, Darcy Road, Westmead, New South Wales, Australia; 5 Department of Infectious Diseases, Peking Union Medical College Hospital, Chinese Academy of Medical Sciences, Beijing, China; 6 Department of Clinical Laboratory, the First Hospital of China Medical University, Shenyang, China; 7 Laboratory of Clinical Microbiology, West China Hospital, Sichuan University, Chengdu, China; 8 Department of Clinical Laboratory, Tongji Hospital, Tongji Medical College, Huazhong University of Science and Technology, Wuhan, China; 9 Department of Clinical Laboratory, Peking University First Hospital, Beijing, China; 10 Department of Clinical Laboratory, the First Affiliated Hospital of Sun Yat-Sen University, Guangzhou, China; 11 Department of Clinical Laboratory, the First Affiliated Hospital of Harbin Medical University, Harbin, China; 12 Department of Clinical Laboratory, the Fourth Affiliated Hospital of Harbin Medical University, Harbin, China

## Abstract

A data analysis of yeast collections from the National China Hospital Invasive Fungal Surveillance Net (CHIF-NET) programme in 2013 revealed a sudden increase in the proportion of *Candida parapsilosis* complex isolates (n = 98) in one participating hospital (Hospital H). Out of 443 yeast isolates submitted to the CHIF-NET reference laboratory by Hospital H (2010–2014), 212 (47.9%) were identified as *C. parapsilosis* sensu stricto by sequencing analysis of the internal transcribed spacer region and D1/D2 domain of the 26S rRNA gene. Among the 212 *C. parapsilosis* sensu stricto isolates, 176 (83.0%) bloodstream-based isolates and 25 isolates from tip cultures of various vascular catheters from 25 patients with candidaemia, were subjected to microsatellite genotyping, and a phylogenetic relationship analysis was performed for 152 isolates. Among the 152 isolates, 45 genotypes (T01 to T45) were identified, and two prevalent genotypes (63.8%) were found: T15 (n = 74, 48.7%) and T16 (n = 23, 15.1%). These two main clones were confined mainly to three different wards of the hospital, and they persisted for 16–25 months and 12–13 months, respectively. The lack of proper coordination between the clinical microbiology laboratory and infection control staff as part of public health control resulted in the failure to timely identify an outbreak, which led to the wide and long-term dissemination of *C. parapsilosis* sensu stricto in Hospital H.

Healthcare-associated infections are important causes of morbidity and mortality among hospitalized patients worldwide. Most nosocomial infections are caused by multidrug-resistant (MDR) bacteria such as methicillin-resistant *Staphylococcus aureus* (MRSA), carbapenem-resistant *Enterobacteriaceae* and *Acinetobacter baumannii*, with outbreaks commonly reported^1^,^2^,^3^,^4^,^5^,^6^. However, nosocomial infections caused by fungi, including outbreaks, have been less frequently reported.

Candidaemia is increasingly being recognized as an important cause of morbidity and mortality in critically ill patients^7^,^8^,^9^. It is generally agreed that candidaemia is an endogenous infection arising from autoinfection after prior colonization of the gastrointestinal tract, skin, or vagina^6^. However, commensalism followed by colonization, which usually precedes dissemination of the organism, may have a nosocomial origin^6^. The majority of nosocomially acquired candidaemia cases are caused by *Candida albicans*^7^, although the emergence of *Candida tropicalis, C. glabrata* and *C. parapsilosis* has also been reported^8^,^9^. *C. parapsilosis* is now considered an important fungal pathogen, being the predominant *Candida* species in bloodstream infections^9^.

*C. parapsilosis* is a significant clinical pathogen that can grow in total parenteral nutrition, can form biofilms on catheters and other implanted devices, can persist in the hospital environment, and may be nosocomially transmitted by hand carriage^10^,^11^,^12^. This organism is the main *Candida* species responsible for a significant proportion of outbreaks of nosocomial fungaemia, particularly in neonate intensive care units (NICUs)^13^,^14^,^15^,^16^,^17^,^18^. However, there are limited studies exclusively assessing the epidemiology of candidaemia caused by *C. parapsilosis* in China, and no description of *C. parapsilosis* clustering or outbreak cases has been reported to date^19^.

The National China Hospital Invasive Fungal Surveillance Net (CHIF-NET) programme is a nationwide, multicentre surveillance network established in July 2009 to provide up-to-date information on the epidemiology of invasive fungal infections in China^20^,^21^. An analysis of the data obtained in the CHIF-NET programme for the years 2010–2014 revealed an increase in the proportion of *C. parapsilosis* sensu stricto isolates in one particular hospital (Hospital H). This increase was particularly marked in the years 2013 and 2014, in which the proportion of *C. parapsilosis* sensu stricto isolates (among all yeasts) was much higher than the annual average level in the past three years, as well as that of other CHIF-NET participating hospitals. This finding prompted us to carry out an in-depth analysis and study of *C. parapsilosis* sensu stricto isolates from Hospital H.

The purpose of this study was to investigate the microbiological characteristics of *C. parapsilosis* sensu stricto isolates collected under the CHIF-NET programme from September 2009 to August 2014, and to analyse the possible clonal transmission of the *C. parapsilosis* sensu stricto in one hospital (Hospital H).

## Results

### Identification of isolates

Consecutive yeast isolates (n = 443) recovered from hospitalized patients with invasive yeast infection at Hospital H (August 2009 to July 2014) were submitted to the CHIF-NET reference laboratory, namely Peking Union Medical College Hospital (PUMCH). Based on the identification results obtained using a commercial phenotypic identification method (Vitek 2 Compact YST card) at the Clinical Microbiology Laboratory of Hospital H, there were 186 (42.0%) *C. parapsilosis* complex isolates and 257 isolates of non-*C. parapsilosis* complex yeasts. A detailed analysis of the distribution of *C. parapsilosis* complex isolates from Hospital H for the period 2010–2014 showed a sharp increase in the proportion of these isolates over the years, ranging from 13.2% (9/68) in 2010 to as high as 65.3% (98/150) in 2013 and 57.0% (53/93) in 2014 (*P* < 0.001).

**Figure 1:**
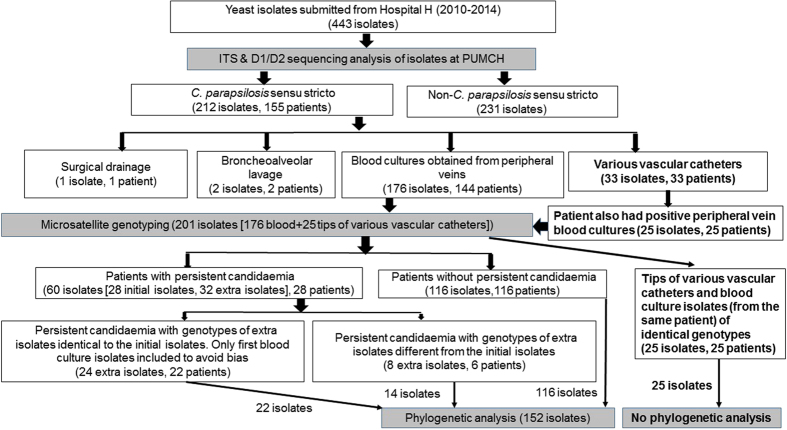
Flowchart of species identification, microsatellite genotyping, and phylogenetic analysis of yeast isolates collected from Hospital H in the CHIF-NET programme from 2010 to 2014. All the 443 yeast isolates collected from Hospital H were further identified to species levels by using sequencing analysis of the internal transcribed spacer region and D1/D2 domain of the 26S rRNA gene at PUMCH. The 33 isolates of *Candida parapsilosis* sensu stricto yielded from tip cultures of various vascular catheters were collected from 33 patients. Among the 33 patients, 25 had positive culture of same yeast species from tip of various vascular catheters as well as from blood samples collected from peripheral veins. Microsatellite genotyping was also performed for the 25 isolates from various vascular catheter tips to demonstrate the possibility of catheter-related candidaemia. Genotypes of isolates recovered from various vascular catheter tips and peripheral veins in each of the 25 patients were identical. Among the 60 *C. parapsilosis* sensu stricto isolates recovered from 28 patients with persistent candidaemia (32 extra isolates in addition to the 28 initial blood isolates), 24 were recovered from 22 patients (two patients with three episodes of candidaemia [2 extra isolates]) with persistent candidaemia showed similar genotypes as the initial culture isolates. Thus to avoid bias, only the 22 first blood culture isolates of the 22 patients were included further phylogenetic analysis. The remaining eight *C. parapsilosis* sensu stricto isolates from six patients with persistent candidaemia (two patients with three episodes of candidaemia [2 extra isolates]) exhibited different genotypes to those of the initial blood culture isolates. These 8 extra isolates and the six first isolates of the six patients (n = 14) were all included in the phylogenetic analysis. As a result, the final number of *C. parapsilosis* sensu stricto isolates subjected to microsatellite genotyping and phylogenetic analysis was 201 and 152, respectively.

After species confirmation (at the reference laboratory) by sequencing analysis of the internal transcribed spacer (ITS) region and D1/D2 domain of the 26S rRNA gene for the 443 yeast isolates from Hospital H, 212 (47.8%) isolates were identified as *C. parapsilosis* sensu stricto (Fig. 1). The remaining 231 non-*C. parapsilosis* sensu stricto species isolates included 15 yeast species, mainly *C. albicans* (n = 98), *C. glabrata* complex (n = 32), *Cryptococcus neoformans* complex (n = 31), *C. tropicalis* (n = 30), and *Rhodotorula mucilaginosa* (n = 15). The identification results for the 443 yeast isolates by sequencing analysis of the ITS region and the D1/D2 domain of the 26S rRNA gene were identical.

Of the 186 isolates identified initially as the *C. parapsilosis* complex at Hospital H, 182 (97.8%) were confirmed by ITS sequencing to be *C. parapsilosis* sensu stricto, one as *C. metapsilosis* (no *C. orthopsilosis* isolate was found), two as *Candida glabrata* sensu stricto, and one as *Cryptococcus laurentii*. Furthermore, among the 257 isolates initially identified as non-*C. parapsilosis* complex at Hospital H, 30 (11.7%) were confirmed as *C. parapsilosis* sensu stricto and four as *C. metapsilosis*, by ITS sequencing. The 212 *C. parapsilosis* sensu stricto isolates were recovered from various clinical specimens (176 from blood cultures collected from peripheral veins, 33 from various vascular catheter tip cultures, two from bronchoalveolar lavage fluid, and one from surgical drainage) of 155 patients (Fig. 1). Various vascular catheters included peripheral venous, central venous, arterial, and peripherally inserted central catheters. The 33 *C. parapsilosis* sensu stricto isolates from tip cultures of various vascular catheters were obtained from 33 patients, 25 of whom had also similar yeast species cultured from peripheral vein blood cultures.

**Figure 2:**
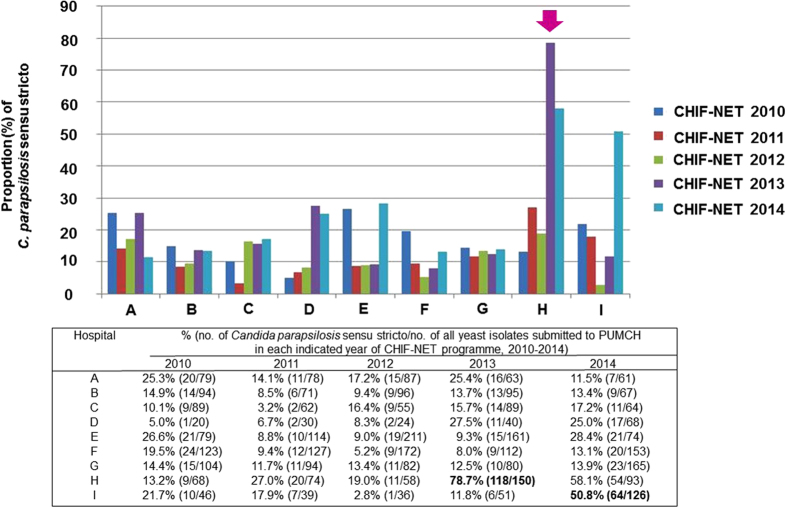
The proportion of *Candida parapsilosis* sensu stricto isolates collected from nine hospitals that consistently participated in the CHIF-NET programme from 2010 to 2014. Of the nine hospitals participating in the CHIF-NET programme during the 5-year study period, the peak proportion of 78.7% at Hospital H (arrow) was noted in 2013 and was significantly higher compared with other hospitals. In the CHIF-NET 2014 programme, the isolation rate of *C. parapsilosis* sensu stricto decreased to 58.1%, but was still higher than those from the years 2010 to 2012. The isolates collected in a specific year of the CHIF-NET programme in an individual hospital were derived from patients treated at the hospital from September 1 of a year to August 31 of the following year. For example, the isolates collected in the CHIF-NET 2010 programme in an individual hospital were those isolated from patients treated at the hospital from September 1, 2009 to August 31, 2010.

An analysis on the distribution of yeast species isolated from Hospital H by year of submission to the CHIF-NET programme showed an obvious increasing trend in the proportion of *C. parapsilosis* sensu stricto isolates (among all yeasts). Specifically, a modest rise was observed from 13.2% (9/68) in 2010 to 27.0% (20/74) and 19% (11/58) in 2011 and 2012, respectively. However, a remarkable rise to 78.7% (118/150) and 58.1% (54/93) was noted in the years 2013 and 2014, respectively (Fig. 2). Furthermore, compared with another eight hospitals that also consistently participated in the CHIF-NET programme, the proportion of *C. parapsilosis* sensu stricto isolates from Hospital H for the year 2013 was significantly higher (78.7% vs. <30.0% for others; *P* < 0.001) (Fig. 2). Similarly, in another hospital (Hospital I), there was a significant rise in the proportion of *C. parapsilosis* sensu stricto isolates (among all yeasts), from 11.8% (6 of 51) in 2013 to 50.8% (64 of 126) in 2014 (*P* < 0.001).

An overwhelming majority (176, 83.0%) of the 212 *C. parapsilosis* sensu stricto isolates from Hospital H were obtained from blood cultures of 144 hospitalized patients with candidaemia. This cohort of isolates was included in the genotyping study. In addition, 25 isolates recovered from tip cultures of various vascular catheters of 25 of the 144 patients with candidaemia, were also subjected to genotyping in order to investigate possible catheter-related candidaemia (Fig. 1). Using the Vitek 2 yeast identification card, the 176 isolates were identified at the Clinical Microbiology Laboratory of Hospital H as *C. parapsilosis* complex (151/176; 85.8%), *Candida famata* (22/176; 12.5%), *C. glabrata* complex (2/176; 1.1%) and *C. laurentii* (1/176; 0.6%).

The 144 hospitalized patients with candidaemia were composed of 80 males and 64 females, with an average age of 67 years (Table 1). These patients were admitted in 13 different wards, and 123 (85.4%) stayed in three main wards: a 126-bed general surgery ward (with three separate locations; 47 patients; 47 isolates), a 42-bed general intensive care unit (ICU) (with three separate locations; 41 patients; 43 isolates), and a 120-bed very important person (VIP) ward (10 different floors; 35 patients; 40 isolates). More than 90% of patients admitted to the VIP ward were elderly (>80 years old).

### Microsatellite genotyping and phylogenetic analysis

Microsatellite genotyping was performed on 201 *C. parapsilosis* sensu stricto isolates, including 176 from peripheral venous blood cultures, and the 25 recovered from tip cultures of various vascular catheters of patients who also had positive blood cultures of the same yeast species. The bloodstream-based isolates included all the initial (first) blood culture isolates from each of the 144 patients (144 isolates; 81.8%) and an extra 32 (18.2%) isolates from 28 patients with persistent candidaemia (defined as repeated isolation of *C. parapsilosis* sensu stricto from blood cultures with a time interval of >7 days). The 28 patients (from the 144 patients) had at least one extra isolate apart from the initial blood culture isolate.

**Figure 3:**
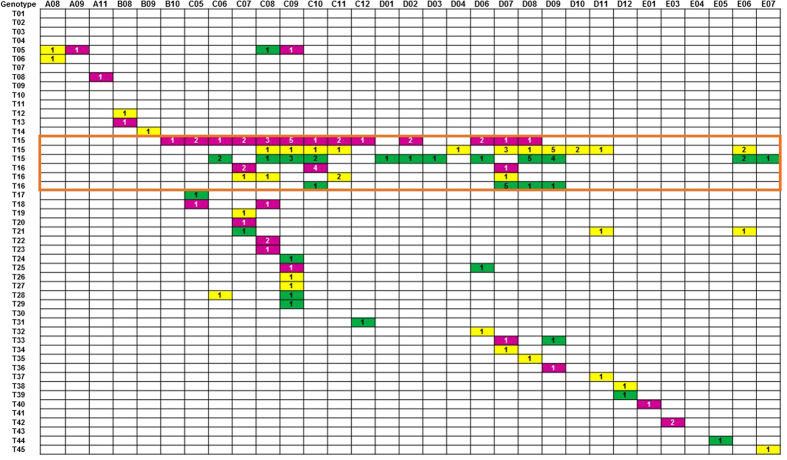
Timeline of the distribution of genotypes of 130 isolates of Candida parapsilosis sensu stricto, time of isolation of these isolates and the three main locations for hospitalized patients in Hospital H. A to E indicates the year of the isolation of *C. parapsilosis* sensu stricto: A, 2010; B, 2011; C, 2012; D, 2013; and E, 2014. The numbers 1 to 12 followed by letters A to E represent months from January to December. Three coloured boxes denote the locations of patients’ hospitalization: green, general surgery ward; yellow, general intensive care unit (ICU); purple, VIP ward. The numbers in the boxes denote the number of isolates detected in the indicated month of the year. The orange square indicates isolates with genotypes T15 and T16.

The 201 isolates were discriminated into 45 different genotypes, which were designated as genotypes T01 to T45. The genotypes of the 25 isolates from various vascular catheter tips were identical to those of peripheral venous blood cultures in each of the 25 patients, and were thus excluded from further phylogenetic analysis to avoid bias. The majority (24/32, 75.0%) of the extra isolates recovered from 22 patients with persistent candidaemia (two patients with three episodes of candidaemia) showed similar genotypes as the initial blood culture isolates. Thus to avoid bias, the genotype results of these 24 isolates were excluded from further phylogenetic analysis; only the first blood culture isolates of these 22 patients were included. The remaining eight *C. parapsilosis* sensu stricto isolates from six patients with persistent candidaemia (two patients had three episodes of candidaemia) exhibiting different genotypes to those of the initial blood culture isolates were included in the phylogenetic analysis. Therefore, 152 *C. parapsilosis* sensu stricto isolates (144 isolates from initial blood culture and eight isolates from six patients with persistent candidaemia) from 144 patients were included for phylogenetic analysis (Table 2 and Fig. 1.), with 80.9% (n = 130) of the isolates concentrated in three main wards (general surgery, general ICU, and VIP wards) (Fig. 3).

Of the 45 genotypes identified, genotypes T15 (n = 74, 48.7%) and T16 (n = 23, 15.1%) were the most prevalent (63.8%), followed by genotype T05 (n = 5, 3.3%). A further seven genotypes, each composed of two to three isolates, were also detected, whereas the remaining 35 genotypes comprised one isolate each (Table 2).

**Figure 4:**
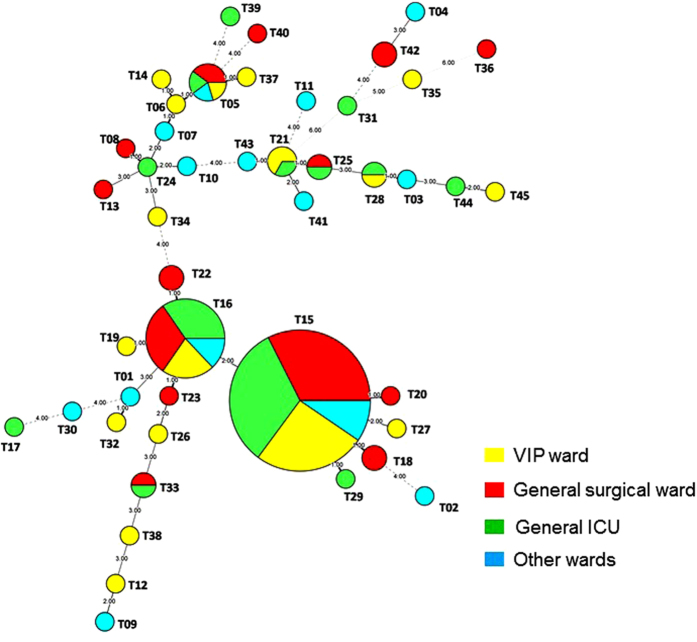
Minimum spanning tree showing the differences between the 45 genotypes among the 152 isolates based on categorical analysis. Each circle represents a unique genotype. The size of the circle corresponds to the number of isolates of the specific genotype. Origin of the samples: green, General surgery ward; yellow, General ICU; red, VIP ward; white, other wards, including medical oncology, neurosurgery, and urinary surgery wards.

Further analysis on the distribution of the predominant genotype (T15) revealed that it first appeared in October 2011, peaked (n = 5) in September 2012, and persisted until August 2013 in the general surgical ward (Fig. 3). In the general ICU ward, genotype T15 first appeared in June 2012, peaked (n = 5) in October 2013, and disappeared in November 2013 (Fig. 3). In the VIP ward, genotype T15 was first noted in August 2012 and persisted until June 2014. For the second most predominant genotype (T16), the highest number of isolates was observed in October 2012 (n = 4) in the general surgical ward and in July 2013 (n = 5) in the general ICU ward. In summary, genotype T15 strains persisted for 16 months in the general ICU, 24 months in the VIP ward, and 25 months in the general surgical ward. By contrast, genotype T16 strains persisted for 12 to 13 months in the general ICU, VIP ward, and general surgical ward. Other genotypes were distributed sporadically by year and ward in the five-year study period (Fig. 3). The two predominant genotypes also only displayed very little variation in the minimum spanning tree (Fig. 4).

### Antifungal susceptibility testing

All of the *C. parapsilosis* sensu stricto isolates were susceptible to fluconazole and voriconazole.

### A literature review on outbreaks caused by *C. parapsilosis* complex

There were 13 reports including the present one on clusters or outbreaks caused by the *C. parapsilosis* complex, and these are summarized in Table 3^13–18,22–27^. The majority of these reports (6; 46.2%) described clusters or outbreaks in NICUs, followed by two in paediatric wards, and the remaining four in adult patients. Only five studies reported on the simultaneous characterization of isolates recovered from patients as well as the hands of healthcare workers (HCWs). The remaining eight studies retrospectively investigated the isolates derived from patients only. Microsatellite genotyping was applied in seven of the studies. The duration of the outbreaks ranged from two weeks to nine years, and the source of the outbreak could be identified in five studies, and it was mostly associated with colonized HCWs’ hands. *C. parapsilosis* sensu stricto was identified as the cause of the outbreak in eight studies by either sequencing analysis of the ITS region and/or the D1/D2 domain of the 26S rRNA gene, and unspecified *C. parapsilosis* complex isolates were reported in the remaining five studies.

## Discussion

Among the *Candida* species causing healthcare-associated outbreaks, the *C. parapsilosis* complex is of remarkable importance due to its propensity to easily colonize human skin (*e.g*., the hands of HCWs), adhere to medical devices, and contaminate hospital environments. These characteristics facilitate the easy spread of the organism in hospital environments, resulting in healthcare-associated outbreaks^10^. Therefore, rapid identification of *C. parapsilosis* complex isolates to the species level and timely investigation on the clonality of isolates can contribute to effective prevention and control of outbreaks and infections caused by this organism^13^,^14^,^15^,^16^,^17^,^18^,^22^,^23^,^24^,^25^,^26^,^27^. However, *C. parapsilosis* complex strains are less virulent than *C. albicans*, *C. tropicalis* and *C. glabrata*, and they are responsible for relatively lower mortality levels among patients with candidaemia^28^. Consequently, infection control practitioners tend to pay less attention and vigilance to potential *C. parapsilosis* complex outbreaks. This lack of attention is in contrast to what normally occurs with potential outbreaks of MDR bacteria or more virulent *Candida* species in many hospitals in China, where there is greater awareness and vigilance of these well-recognized infectious agents^2^,^4^,^5^,^28^.

This microbiological investigation of *C. parapsilosis* complex isolates from a single hospital (Hospital H) from 2010 to 2014 revealed several important points. First, the lack of early warning systems from laboratory staff and infection control practitioners for the increasing number of *C. parapsilosis* complex isolates (the occurrence of more cases of disease than expected) in early 2012 clearly contributed to the wide and long-term dissemination of candidaemia due to *C. parapsilosis* sensu stricto in the hospital. This finding highlights the need to initiate and strengthen epidemiological data collection systems for monitoring the spread of organisms between the laboratory and infection control systems in hospitals in China. This situation was not only observed in one hospital (Hospital H), but it appears to also have occurred at another hospital (Hospital I), where the proportion of *C. parapsilosis* sensu stricto isolates rose from 11.8% in 2013 to 50.8% in 2014 (Fig. 1). Unfortunately, the way the CHIF-NET programme is structured hindered the timely communication of vital information from the central laboratory to the laboratory staff and infection control practitioners at Hospital H about the potential *C. parapsilosis* sensu stricto outbreak. Specifically, the CHIF-NET yearly programme runs from September of one year to the end of August the following year, with all the collected isolates in the preceding year submitted to the central laboratory once in early September. This means that in 2013, the CHIF-NET central laboratory only noted the increased proportion of *C. parapsilosis* complex isolates from Hospital H some time later in September, which was one year later.

Second, this study shows that an outbreak of candidaemia due to *C. parapsilosis* sensu stricto most likely occurred at Hospital H. This conclusion is supported by the fact that, with the exception of five isolates (*C. metapsilosis*), all of the *C. parapsilosis* complex isolates collected over the 5-year study period were confirmed as *C. parapsilosis* sensu stricto by ITS sequencing. Furthermore, using microsatellite typing, two main strains (genotypes T15 and T16) that accounted for more than 60% of all *C. parapsilosis* sensu stricto isolates, were concentrated in three different wards in Hospital H. Microsatellite genotyping has emerged as the most commonly used and versatile method for typing pathogenic fungi, especially for *C. parapsilosis* complex isolates, which, given the clonal nature of this organism, cannot be typed by multilocus sequencing analysis, restriction fragment length polymorphism, karyotyping, and/or random amplified polymorphic DNA analysis^17^. The fact that genotype T15 and T16 strains could persist for 16–25 months and 12–13 months, respectively, mainly in three wards (general surgical ward, general ICU, and VIP ward) of the hospital suggests that two main clones of *C. parapsilosis* sensu stricto were responsible for the unrecognized outbreak in Hospital H.

Third, in contrast to previous outbreaks of the *C. parapsilosis* complex mostly confined to NICUs, the majority of the patients in the present study were adults, including elderly people, and they were confined to three wards, namely the surgical, general ICU, and VIP wards^13^,^14^,^15^,^16^,^17^,^18^. Finally, to the best of our knowledge, this is the first study to report on a candidaemia outbreak (and with the largest number of isolates) due to *C. parapsilosis* sensu stricto in China.

In the present study, isolates from 25 patients with simultaneous positive cultures of *C. parapsilosis* sensu stricto from peripheral blood samples and tip cultures of various vascular catheters exhibited the same genotypes, indicating that catheters may serve as an important transmission source for this organism. Indeed, several studies have demonstrated that indirect transmission of the *C. parapsilosis* complex via the hands of HCWs is responsible for outbreaks of catheter-related *C. parapsilosis* infections in NICUs^10^,^13^,^28^,^29^. However, a study by Delfino *et al.* reported that genotypes of *C. parapsilosis* strains from the hands of HCWs exhibited considerable heterogeneity and showed higher genetic diversity than those from patients with candidaemia^30^. Other possible sources of infection amongst patients may also be related to the well-known risk factors in a hospital environment, including contaminated medical devices and environmental surfacs^10^,^13^,^28^,^29^. Nevertheless, this assumption remains purely speculative as no cultures of the hands of HCWs or the hospital environment were performed as part of this retrospective investigation.

This study has three main limitations. First, the clinical characteristics, risk factors, and outcomes of patients with *C. parapsilosis* sensu stricto candidaemia and those associated with the outbreak were not evaluated, as this study was performed retrospectively. Second, although the number of *C. parapsilosis* sensu stricto isolates collected from Hospital H declined in 2014, the precise time the outbreak declined remains unknown and warrants further investigation. Third, only bloodstream-derived *C. parapsilosis* sensu stricto isolates were genotyped in this study. It is possible that strains of dominant genotypes might also be found in various clinical specimens of patients without candidaemia in Hospital H. Thus, further work is needed to investigate the clinical and microbiological consequences of the event at Hospital H, including the possible emergence of a similar outbreak in other hospitals (*e.g.*, Hospital I).

A lack of awareness of healthcare-associated infections and outbreaks, particularly those of fungal origin, among laboratory staff and infection control practitioners is common in major hospitals in China. Thus, continued public health efforts aimed at education, training, defining, characterizing, and tracking the emergence of fungal infections and possible outbreaks is needed to help focus studies on priority infections and settings.

In conclusion, the lack of timely alertness from laboratory staff and infection control practitioners to the increasing number of *C. parapsilosis* complex isolates clearly contributed to the failure to recognize a *C. parapsilosis* complex outbreak, resulting in wide and long-term dissemination of *C. parapsilosis* sensu stricto candidaemia in the studied hospital. Consequently, an opportunity to investigate the source of the outbreak and implement associated infection control measures was lost. This study offers a lesson and warning to the overall healthcare system in China regarding the need to strengthen the collaboration between the clinical microbiology laboratory and infection control systems as part of the public health control of diseases, and it further raises the challenge to revisit the current infection control strategies for the early recognition and management of outbreaks, not only for well-known MDR bacteria but also for *Candida* species and other fungi.

## Methods

### Background, setting and isolates

Hospital H is a 2,000-bed tertiary-care teaching hospital in northern China, which has participated in the CHIF-NET programme since 2010. As part of the CHIF-NET program, all consecutive yeast isolates from patients with invasive infections, including from blood and other sterile body fluids (ascites, peritoneal dialysate fluid, and surgical drainage/pus) and tissues at Hospital H (2010–2014), were sent to the reference hospital laboratory and were included in this study^20^. Also included were yeast isolates from broncheoalveolar lavage fluid, central venous catheter tips, and the gastrointestinal tract (e.g., aseptically collected bile). Yeast isolates from urine, genital tract, and others that were considered colonizers were excluded. Isolates of the same yeast species from the same site of a given patient that were recovered at different times were considered duplicates and were excluded. However, yeast isolates from persistent fungaemia cases (defined as repeated isolation of same yeast species from blood cultures with a time interval of >7 days) were included^20^.

By the end of August 2014, 443 yeast isolates had been submitted by this hospital to the CHIF-NET reference laboratory at Peking Union Medical College Hospital (PUMCH). The yeast isolates were identified at Hospital H by Vitek 2 yeast identification card (Vitek 2 Compact YST card, bioMérieux Marcy l’Etoile, France). The isolates were identified to the species level by analysing the fungal ITS region and the D1/D2 domain of the 26S rRNA gene at the reference laboratory^31^,^32^. This study was approved by the Human Research Ethics Committee of PUMCH (No. S-263). No written informed consents were obtained from patients as the study did not include patient details, images or videos. All procedures and methods were carried out in accordance with approved guidelines.

### Identification of the isolates at the central laboratory

The identity of the 443 yeast isolates was confirmed by amplification of the fungal ITS region and the D1/D2 domain of the 26S rRNA gene, as previously described using primer pairs ITS1/ITS4 and F63/R635, respectively^31^,^32^. Briefly, all of the isolates were inoculated onto Brilliance *Candida* agar (Oxoid Ltd.) and then subcultured on Sabouraud dextrose agar (Thermo Fisher Scientific, MA, USA). Culture media were incubated for 24–48 h at 35 °C. A pure colony of the organism was picked using a 1 μl loop and mixed with 50 μl of glass beads and 400 μl of distilled water in an Eppendorf tube. After vortexing for 1.5 min, the tube was placed in boiling water (100 °C) for 15 min. Then, the tube was placed at −20 °C for 10 min, followed by centrifugation at 12,000 rpm for 5 min. The pellet was used for sequencing analysis.

For ITS sequencing, primers ITS1 (5′-TCCGTAGGTGAACCTGCGG-3′) and ITS4 (5′-TCC TCCGCTTATTGATATGC-3′) were used^31^. As previously described, primers F63 (5′-GCATATCAATAAGCGGAGGAAAAG-3′) and R635 (5′-GGTCCGTGTTTCAAGACG-3′) were used for D1/D2 region sequencing^32^. Amplification reactions for the ITS and D1/D2 region were performed as previously reported^20^,^21^. In brief, ITS region amplification was carried out under the following conditions: denaturation at 94 °C for 10 min, followed by 30 cycles of denaturation at 94 °C for 30 s, annealing at 50 °C for 30 s, and elongation at 72 °C for 60 s, with a final extension step of 10 min at 72 °C. Amplification conditions for the D1/D2 region included an initial denaturation of 4 min at 94 °C, followed by 18 cycles of 94 °C for 30 s, annealing at 62 °C for 45 s, and elongation at 72 °C for 60 s, with a final extension at 72 °C of 4 min. The obtained sequences were then compared with those available in GenBank using the BLAST program (http://blast.ncbi.nlm.nih.gov/Blast.cgi). The strategy for accurate ITS sequence-based identification of human and animal pathogenic fungi, recommended by the ISHAM (http://its.mycologylab.org/), was followed^33^. The results were considered acceptable if the homology with other entries in the databases used for comparison was >99.5%^33^.

### Antifungal susceptibility testing

Antifungal susceptibility to fluconazole (25 μg disc) and voriconazole (1 μg disc) (Beckon Dickinson, Sparks, MD, USA) was determined for all isolates using the CLSI M44-A2 disc diffusion method and the results were interpreted according to CLSI standards^34^,^35^. For each run, the quality control strains were *C. parapsilosis* ATCC 22019 and *C. krusei* ATCC 6258.

### Microsatellite typing

Yeast isolates were genotyped using four highly polymorphic microsatellite markers described by Sabino *et al.*, namely B5, CP1, CP4 and CP6^36^. The forward primers were labelled with either 6-carboxyfluores-cein or hexachlorofluorescein. Amplification reactions were performed as previously reported^18^. Following PCR, amplicons were sized by capillary electrophoresis on an ABI 3730XL DNA Analyzer (Applied Biosystems, Foster City, CA, USA) coupled with GeneMarker v1.8 software (SoftGenetics LLC, State College, PA, USA). Allele sizes were scored with respect to GeneScan™ 500 LIZ^®^ Size Standard (Applied Biosystems) in the 35–500 nt range.

### Phylogenetic relationship analysis

The relationship between genotypes was visualized by constructing a minimum spanning tree using the online tool Tree Drawing (http://www.pubmlst.org), treating the data as categorical information.

### Statistical analysis

Annual proportions of isolation of the *C. parapsilosis* complex or *C. parapsilosis* sensu stricto among all yeast isolates over time in Hospital H were evaluated with the Cochran-Armitage test for trend. A *P* value < 0.05 was considered to be significant.

### Literature review of the clusters or outbreaks caused by *C. parapsilosis* complex

We searched the PubMed database (http://www.ncbi.nlm.nih.gov/pubmed/) and the US Centers for Disease Control and Prevention website (http://wwwnc.cdc.gov/eid/) with key words of “*Candida parapsilosis*” combined with “outbreak” or “clustering”, and we limited the search to studies conducted from January 2000 to December 2014. The results are summarized in Table 3^13–18,22–27^.

## Additional Information

**How to cite this article**: Wang, H. *et al.* Investigation of an unrecognized large-scale outbreak of *Candida parapsilosis* sensu stricto fungaemia in a tertiary-care hospital in China. *Sci. Rep.* **6**, 27099; doi: 10.1038/srep27099 (2016).

## Figures and Tables

**Table 1:** Demographic data of patients.

Patient characteristics	Variable	No. (%) of patients hospitalized in each indicated hospital setting				
		General surgery ward	General ICU	VIP ward	Other wards^a^	Total
Sex	Male	26 (55.3)	23 (56.1)	25 (71.4)	6 (28.6)	80 (55.6)
	Female	21 (44.7)	18 (43.9)	10 (28.6)	15 (71.4)	64 (44.4)
Age	<20	1 (2.1)	1 (2.4)	0	0	2 (1.4)
	21–40	6 (12.8)	4 (9.8)	0	3 (14.3)	13 (9.0)
	41–60	17 (36.2)	11 (26.8)	1 (2.9)	5 (23.8)	34 (23.6)
	61–80	21 (44.9)	21 (51.2)	1 (2.9)	7 (33.3)	50 (34.7)
	>80	2 (4.3)	4 (9.8)	33 (94.3)	6 (28.6)	45 (31.3)
No. of patients	All age groups	47	41	35	21	144

The table shows the sex and age groups of 144 hospitalized patients with candidaemia due to *Candida parapsilosis* sensu stricto in Hospital H.

^a^Other wards include medical oncology, neurosurgery, and urinary surgery wards ICU, intensive care unit; VIP, very important person.

**Table 2:** Designations of the 45 genotypes.

Genotype	Designation of microsatellite markers								No. (%) of isolates
	B5-a	B5-b	CP-1-a	CP-1-b	CP-4-a	CP-4-b	CP-6-a	CP-6-b	
T01	129	129	246	246	372	390	306	306	1 (0.7)
T02	127	129	210	246	327	363	225	309	1 (0.7)
T03	113	129	243	246	366	366	273	273	1 (0.7)
T04	109	109	210	243	279	309	291	291	1 (0.7)
T05	129	129	243	246	369	411	237	282	5 (3.3)
T06	129	149	243	246	369	411	237	282	1 (0.7)
T07	129	149	243	246	369	369	237	282	1 (0.7)
T08	129	143	243	246	369	369	282	303	1 (0.7)
T09	127	129	243	261	357	357	267	267	1 (0.7)
T10	129	131	243	246	369	369	300	303	1 (0.7)
T11	129	131	243	243	372	390	276	288	1 (0.7)
T12	129	129	246	261	357	357	267	267	1 (0.7)
T13	129	129	228	246	369	369	282	306	1 (0.7)
T14	129	149	246	246	369	411	237	282	1 (0.7)
T15	127	129	246	246	396	396	303	306	74 (48.7)
T16	127	129	246	246	372	375	303	306	23 (15.1)
T17	113	113	246	246	360	360	273	282	1 (0.7)
T18	127	129	246	246	396	396	303	309	2 (1.3)
T19	127	129	246	246	372	393	303	306	1 (0.7)
T20	127	129	246	246	396	396	303	324	1 (0.7)
T21	129	131	243	243	366	366	303	330	3 (2.0)
T22	127	129	246	246	375	375	303	306	2 (1.3)
T23	127	129	246	246	372	372	303	306	1 (0.7)
T24	129	149	243	246	369	369	282	303	1 (0.7)
T25	129	129	243	243	366	366	303	330	2 (1.3)
T26	129	129	225	246	372	372	303	306	1 (0.7)
T27	127	127	246	246	396	396	306	306	1 (0.7)
T28	113	129	243	243	366	366	273	273	2 (1.3)
T29	127	129	246	246	396	399	303	306	1 (0.7)
T30	129	129	246	246	360	360	231	273	1 (0.7)
T31	127	145	243	243	306	306	279	279	1 (0.7)
T32	129	129	246	246	390	390	306	306	1 (0.7)
T33	129	129	225	246	357	384	303	336	2 (1.3)
T34	129	129	243	246	375	375	282	303	1 (0.7)
T35	127	145	222	243	303	303	249	249	1 (0.7)
T36	121	121	243	252	303	303	264	264	1 (0.7)
T37	129	129	243	246	369	411	237	237	1 (0.7)
T38	129	129	246	246	357	384	267	270	1 (0.7)
T39	129	129	243	243	369	372	288	303	1 (0.7)
T40	129	129	243	246	399	402	297	297	1 (0.7)
T41	129	131	243	246	366	366	303	306	1 (0.7)
T42	109	109	243	243	306	306	291	291	2 (1.3)
T43	129	131	243	243	366	366	303	303	1 (0.7)
T44	113	129	243	246	366	396	267	285	1 (0.7)
T45	127	129	243	246	366	366	267	285	1 (0.7)

The table shows the 45 genotypes (T01–T45) among 152 non-repetitive *C. parapsilosis* sensu stricto isolates generated by microsatellite genotyping.

**Table 3:** Literature review of outbreaks or clusters due to *Candida parapsilosis* complex.

No.	Year of report	Country	Ward or ICU	No. and sources of isolates (patients/HCWs/environment)	Duration	Identification of possible source of outbreak	*C. parapsilosis*	Genotyping method (s)	Author [reference]
1.*	2010	Mexico	NICU	6/2/0	NA	Yes (HCW’s hand)	sensu stricto	RAPD	Hernandez-Castro *et al.*^13^
2.*	2011	Portugal	NICU	28/7/0	NA	Yes (HCW’s hand)	sensu stricto	Microsatellite	Vaz *et al.*^14^
3.	2012	Brazil	NICU	36/71/0	7 y	Yes (HCW’s hand)	sensu stricto	PFGE, RAPD	Miranda *et al.*^15^
4.	2012	Italy	NICU	19/0/0	4 y	No	complex	Microsatellite, PFGE MALDI-TOF MS	Pulcrano *et al.*^16^
5.	2012	USA	NICU	64/0/0	3.5–9 y	No	sensu stricto	Microsatellite	Reiss *et al.*^17^
6.	2013	Italy	NICU	27/0/0	4 y	No	sensu stricto	Microsatellite	Romeo *et al.*^18^
7.	2004	Italy	Pediatric oncology unit	27/3/1	4 m	Yes (HCW’s hand)	complex	Electrophoretic karyotyping	Barchiesi *et al.*^22^
8.	2004	Spain	Pediatric ICU	9/0/0	2 y	No	complex	Electrophoretic karyotyping, RAPD	Garcia *et al.*^23^
9.*	2004	the United States	NA	5/3/0	10 m	Yes (HCW’s hand)	complex	DNA fingerprinting	Kuhn *et al.*^24^
10.	2008	Turkey	Neurological ICU (adults)	4/0/0	2 w	No	sensu stricto	RAPD	Dizbay *et al.*^25^
11.	2009	Sweden	Haematology ward	4/0/0	1 m	No	complex	Microsatellite, RAPD	Brillowska-Dabrowska *et al.*^26^
12.*	2012	Austria	Cardiothoracic SICU	99/0/0	2 y	No	sensu stricto	Microsatellite, automated repetitive sequence based PCR	Diab-Elschahawi *et al.*^27^
13	2010–2014	China	General surgery ward, general ICU, VIP ward	152/0/0	4 y	No	sensu stricto	Microsatellite	Wang *et al.* [present report]

The table summarizes 13 outbreaks or clusters (including the present study) due to the presence of the *C. parapsilosis* complex in neonatal ICU (no. 1–6) and other hospital settings (no. 7–12) and that in the present report (no. 13).

HCW, healthcare worker; ICU, intensive care unit; MALDI-TOF MS, Matrix-Assisted Laser Desorption/Ionization Time-of-Flight Mass Spectrometry; NA, not available; NICU, neonate ICU; PFGE, pulsed-field gel electrophoresis; RAPD, randomly amplified polymorphic DNA; SICU, surgical ICU; VIP, very important person.

^*^Indicates a prospective study, otherwise a retrospective study.
